# Assessment of the amino acid profile in Thai patients with type 2 diabetes mellitus using liquid chromatography-mass spectrometry

**DOI:** 10.1093/inthealth/ihaa083

**Published:** 2020-10-28

**Authors:** Natthida Sriboonvorakul, Wirichada Pan-Ngum, Kittiyod Poovorawan, Markus Winterberg, Joel Tarning, Sant Muangnoicharoen

**Affiliations:** Department of Clinical Tropical Medicine, Faculty of Tropical Medicine, Mahidol University, Bangkok, Thailand; Department of Tropical Hygiene, Faculty of Tropical Medicine, Mahidol University, Bangkok, Thailand; Department of Clinical Tropical Medicine, Faculty of Tropical Medicine, Mahidol University, Bangkok, Thailand; Mahidol Oxford Tropical Medicine Research Unit, Faculty of Tropical Medicine, Mahidol University, Bangkok, Thailand; Centre for Tropical Medicine, Nuffield Department of Clinical Medicine, University of Oxford, Oxford, United Kingdom; Mahidol Oxford Tropical Medicine Research Unit, Faculty of Tropical Medicine, Mahidol University, Bangkok, Thailand; Centre for Tropical Medicine, Nuffield Department of Clinical Medicine, University of Oxford, Oxford, United Kingdom; Department of Clinical Tropical Medicine, Faculty of Tropical Medicine, Mahidol University, Bangkok, Thailand

**Keywords:** amino acids, global health, mass spectrometry, type 2 diabetes mellitus

## Abstract

**Background:**

Type 2 diabetes mellitus (T2DM) is a global health problem. Early identification of those at risk is necessary to prevent its onset through lifestyle and pharmacologic interventions. T2DM is characterized by metabolic abnormalities, including protein metabolism. Evaluation of the amino acid profile might be beneficial for early assessment.

**Methods:**

Liquid chromatography-mass spectrometry was performed to separate and quantify plasma amino acids from two groups of Thai individuals, patients with T2DM (n=103) and healthy individuals (n=104). Multivariate analysis was applied to compare free amino acid levels between groups. Subgroup analyses of patients with T2DM were performed to assess the association between amino acid profiles and important T2DM clinical characteristics.

**Results:**

The multivariate analysis showed that glutamic acid was significantly associated with T2DM (OR 1.113, 95% CI 1.006 to 1.231) and results from the subgroup analyses showed that this correlation was significant in all subgroups of patients (p<0.05).

**Conclusions:**

This finding needs to be confirmed in larger groups of patients with T2DM to explore glutamic acid as a biomarker for early prevention in particular at-risk groups. An in-depth understanding of the involvement of glutamic acid in T2DM could enhance our understanding of the disease and potentially provide novel interventions.

## Introduction

Type 2 diabetes mellitus (T2DM), a chronic non-communicable disease that leads to various health problems, is a global health issue. It is the most common form of diabetes and is generally characterized by adult onset and insulin resistance.^1^ Because of hyperglycemia and insulin resistance, individuals with T2DM are at high risk of both microvascular complications (including neuropathy, retinopathy and nephropathy) and macrovascular complications (such as stroke and heart attack).^2^

Currently, routine testing for T2DM is normally performed by the measurement of fasting plasma glucose (FPG) or hemoglobin A1c (HbA1c). However, in many cases these two forms of testing fail to detect early T2DM, which is also called pre-diabetes. Ultimately, early identification of those at risk of T2DM is required in the elucidation of its molecular etiology and for delaying T2DM manifestation by lifestyle and pharmacologic interventions.^3^

T2DM is not only characterized by glucose intolerance but also by dysregulation of protein metabolism, which results from impaired insulin secretion and/or insulin resistance.^4^ In the case of energy deficiency, proteins provide an alternative energy source. Deficiency of insulin (the key hormone in glucose metabolism) leads to increased gluconeogenesis, glycogenolysis and protein breakdown in skeletal muscle.^5^ Amino acids are the dynamic structural building blocks of proteins and are active signaling molecules regulating metabolism.^6^ In particular, in the case of insulin resistance (a characteristic for diabetes), proteolysis is increased whereas protein synthesis is decreased.^7^ Increased concentrations of plasma amino acids are currently suggested as predicting the risk of T2DM and therefore amino acid profiling could assist in better understanding the metabolism pathways related to T2DM.^8^ Therefore, evaluation of the amino acid profile could be of benefit for early assessment of the risk of developing T2DM.

Analysis of metabolites, in particular amino acids, in body fluids has been a crucial part of the diagnosis, prognosis and assessment of therapeutic interventions in clinical applications.^9^ The 12-y Framingham Offspring study demonstrated that branched chain amino acids (BCAAs) and aromatic amino acids were significantly associated with the future development of T2DM.^8,10^ Plasma amino acid concentrations not only depend on homoeostatic control, but are also affected by diet, metabolism, lifestyle and genetic factors.^11^

A previous study showed that there was no correlation between the amino acid content in the diet and the plasma amino acid level.^12^ The study also compared plasma amino acid levels between different habitual diets and the results showed that amino acid levels were not significantly different between meat eaters, fish eaters, vegetarians or vegans.^12^

In this case-control observational study, we aimed to study two individual Thai groups, patients with T2DM and healthy participants as the control for assessment of the amino acid profile using liquid chromatography-mass spectrometry (LC-MS) for the separation, identification and quantification of amino acids in plasma samples. A subgroup analysis of the patients with T2DM was also performed to assess the association between amino acid profile and important T2DM clinical characteristics.

## Materials and Methods

### Patients, participants and sample collection

Written informed consent was obtained from all the participants in the study. All Thai adult participants were recruited at the Hospital for Tropical Diseases, Faculty of Tropical Medicine, Bangkok, Thailand. Participants were of both genders and aged 35–65 y. Baseline clinical and laboratory data of participants were obtained in the hospital (i.e. glucose, HbA1c and lipid profile). Pregnant women were excluded from the study.

This case-control observational study classified cases (patients with T2DM) by using the modified 2018 WHO criteria,^13^ individuals who had fasting plasma glucose (FPG) ≥126 mg/dL or a history of FPG ≥126 mg/dL. Controls (healthy individuals) were classified by an FPG <100 mg/dL and with no history of elevated FPG. Patients with T2DM were subgrouped by five important clinical characteristics related to T2DM according to the 2014 American Diabetes Association (ADA)^1^ classification and the modified 2018 WHO criteria^13^ for Asian populations.

The first subgroup of patients with T2DM was classified by plasma HbA1c level using the 2014 ADA criteria^1^ of uncontrolled T2DM (uncontrolled T2DM with HbA1c ≥ 0.154 g/dl vs controlled T2DM with HbA1c < 0.154 g/dl) compared with healthy subjects (HbA1c < 0.154 g/dl; no healthy subjects had HbA1c ≥ 0.154 g/dl). The group of healthy subjects with HbA1c < 0.154 g/dl was used as a reference group in the statistical evaluation.

The second subgroup of patients with T2DM was classified by age using the modified 2018 WHO criteria of older patients (patients with T2DM aged ≥60 y vs patients with T2DM aged <60 y) compared with healthy subjects aged ≥60 and <60 y. The group of healthy subjects aged <60 y was used as a reference group in the statistical evaluation.

The third subgroup of patients with T2DM was classified by body mass index (BMI) using the modified 2018 WHO criteria of overweight for an Asian population (patients with T2DM with BMI ≥23 kg/m^2^ vs patients with T2DM with BMI <23 kg/m^2^) compared with healthy subjects (healthy subjects with BMI ≥23 kg/m^2^ and healthy subjects with BMI <23 kg/m^2^). The group of healthy subjects with normal weight (BMI <23 kg/m^2^) was used as a reference group in the statistical evaluation.

The fourth subgroup of patients with T2DM was classified by hypertension using the modified 2018 WHO criteria (patients with T2DM with systolic blood pressure [SBP] ≥140 mmHg and/or diastolic blood pressure [DBP] ≥90 mmHg vs patients with T2DM with SBP <140 mmHg and DBP <90 mmHg) compared with healthy subjects (healthy subjects with SBP ≥140 mmHg and/or DBP ≥90 mmHg, and healthy subjects with SBP <140 mmHg and DBP <90 mmHg). The group of healthy subjects with normal blood pressure (SBP <140 mmHg and DBP <90 mmHg) was used as a reference group in the statistical evaluation.

The fifth subgroup of patients with T2DM was classified by parental history of T2DM, including one or both parents (patients with T2DM with a parental history of T2DM vs patients with T2DM without a parental history of T2DM), compared with healthy subjects with and without a parental history of T2DM. The group of healthy subjects without a parental history of T2DM was used as a reference group in the statistical evaluation.

Venous blood was collected from all participants after fasting for 8 h and then divided into four aliquots. The first aliquot was whole blood collected in an EDTA tube for HbA1c analysis; the second aliquot was collected in a sodium fluoride tube and centrifuged for 7 min at 2500 g at room temperature to prepare plasma for glucose analysis; and the third aliquot was collected in a clot blood tube and centrifuged for 7 min at 2500 g at room temperature to prepare serum for creatinine and lipid profile (cholesterol, triglyceride, high-density lipoprotein and low-density lipoprotein) analyses. All blood chemistry analyses were performed using a Cobas c501 Chemistry analyzer (Roche, Basel, Switzerland) in the hospital laboratory. The fourth aliquot was collected in an EDTA tube and centrifuged for 7 min at 2500 g at room temperature to prepare plasma that was stored at −80°C until analysis of the amino acids by LC-MS.

### Analytical methods

Amino acids were extracted from individual plasma samples for analysis using a PhenomenexEZ: faast amino acid analysis kit (Phenomenex, Torrance, CA, USA) as described previously.^14^

Plasma-free amino acids were separated and quantified by LC-MS using an API 5000 Triple Quadrupole Mass Spectrometer. Reversed-phase chromatographic separation was performed on an EZ: fasstTM AAA-MS column (250 × 3.0 mm, Phenomenex) at 35°C and a flow rate of 250 µl/min with a total run time of 17 min. The mobile phase consisted of an A-B mixture of 10 mM ammonium formate in water (A) and 10 mM ammonium formate in methanol (B), where the methanol percentage was changed linearly as follows: 0 min, 68%; 2.5 min, 83%; 10 min, 83%; and 17 min, 68%. A sample volume of 1 µl was injected and detection was performed using tandem MS with multiple reaction monitoring (MRM) transitions in the positive ion mode with electro-spray ionization. The MRM method was set fixed Q1 and Q3 for mass scanning and consisted of three periods of analysis, which had a total of 20 MRMs. The optimized MRM transitions and retention times for 20 amino acids are summarized in Table S1. Prior to data acquisition, we optimized the LC system to detect the internal standards (homoarginine, methionine-d3, homophenylalanine). The scan range was 50–600 m/z. Inter-assay variation scores were determined for the three amino acid standards. Quantitative data were obtained from calibration standards based on a linear standard curve using MultiQuant (AB SCIEX, Framingham, MA, USA).

### Statistical methods

Data are presented as geometric means (95% CI). Differences between patients with T2DM and healthy participants (controls) were analyzed using the Mann-Whitney rank sum test (continuous data) and Fisher's exact test (proportions). Correlation between variables was evaluated using Spearman’s rank order correlation. Multivariate statistical analysis was performed using multiple logistic regression between patients with T2DM and healthy participants with adjustment for age, gender and BMI.^8^ Furthermore, multinomial logistic regression analysis of the five individual subgroups of patients with T2DM (classified by HbA1c, age, BMI, hypertension and parental history of T2DM) was performed individually in each subgroup to evaluate ORs. Statistical analyses were conducted using GraphPad PRISM version 5.03 (GraphPad Software, San Diego, CA, USA) and SPSS version 18.0 (SPSS, New York, NY, USA). The level of significance was set at p<0.05.

## Results

### Patient and participant characteristics and univariate analysis

Baseline clinical and laboratory characteristics are summarized in Table 1.

**Table 1.** Baseline clinical and laboratory variables

Characteristics	T2DM (n=103)	Healthy (n=104)	p-value
Male, n (%)	40 (38.83%)	19 (18.27%)	0.0012
Age (y)	52 (51–54)	47 (46–49)	<0.0001
Body mass index (kg/m^2^)	28.39 (27.38–29.44)	24.64 (23.90–25.41)	<0.0001
Parental history of diabetes, n (%)	67 (65.05%)	44 (42.31%)	0.0013
Hypertension, n (%)	62 (60.19%)	25 (24.04%)	<0.0001
Fasting glucose (mg/dl)	138.9 (130.5–147.7)	90.20 (88.23–92.22)	<0.0001
HbA1c (g/dl)	0.157 (0.150–0.165)	0.106 (0.103–0.108)	<0.0001
Cholesterol (mg/dl)	182.4 (175.8–189.4)	202.9 (196.4–209.8)	<0.0001
Triglyceride (mg/dl)	136.1 (124.7–148.5)	98.32 (90.52–106.8)	<0.0001
HDL (mg/dl)	52.52 (50.11–55.05)	62.11 (59.49–64.84)	<0.0001
LDL (mg/dl)	95.97 (90.08–102.3)	115.6 (108.7–122.8)	<0.0001
Urine protein, n (%)	11 (10.68)	3 (2.88)	0.0289
eGFR <60 ml/min/1.73m^2^, n (%)	7 (6.80%)	4 (3.85%)	0.3733
Plasma concentration (nmol/L)			
Valine	138.3 (112.1–170.6)	213.1 (196.9–230.6)	0.0009
Leucine	116.1 (99.70–135.2)	196.1 (181.8–211.5)	<0.0001
Isoleucine	149.5 (131.5–170.0)	174.9 (158.2–193.3)	0.0359
Phenylalanine	111.5 (95.36- 130.5)	114.9 (101.6–129.9)	0.8591
Tyrosine	32.75 (28.56–37.56)	50.20 (46.89–53.75)	<0.0001
2-Aminoadipic	2.520 (2.295–2.767)	2.143 (1.922–2.390)	0.0031
Arginine	44.78 (38.70–51.81)	86.89 (78.87–95.71)	<0.0001
Glycine	147.3 (125.3–175.6)	259.4 (212.9–316.1)	<0.0001
Threonine	208.2 (166.9- 259.8)	138.8 (120.8–159.4)	0.0001
Methionine	16.19 (14.83–17.67)	23.97 (22.77–25.24)	<0.0001
Aspatic	6.747 (6.344–7.176)	5.182 (4.852–5.534)	< 0.0001
Sacosine	223.0 (187.6–265.1)	293.6 (268.3–321.3)	0.0181
Ornithine	82.63 (77.28–88.34)	100.5 (93.55–108.0)	<0.0001
Proline	170.1 (148.5–194.8)	176.6 (165.5–188.3)	0.4213
Lysine	160.9 (150.1–172.5)	213.8 (203.1–225.0)	<0.0001
Glutamic	169.6 (134.5–213.9)	147.8 (135.8–160.9)	0.0086
Glutamine	155.3 (133.6–180.5)	250.1 (228.8–273.4)	<0.0001
Serine	166.7 (135.7–204.7)	110.9 (97.34–126.3)	0.0002
Asparagine	65.16 (54.74–77.55)	53.44 (47.65–59.93)	0.0371
Hydroxyproline	21.87 (18.48–25.87)	17.00 (14.48–19.96)	0.0674

Abbreviations: eGFR, estimated glomerular filtration rate; HDL, high-density lipoprotein; LDL, low-density lipoprotein; T2DM, type 2 diabetes mellitus.

All values are presented as geometric mean (95% CI) unless otherwise specified.

Univariate analysis of 20 amino acids between the two groups (patients with T2DM and healthy controls) showed that the concentration of six plasma acids, glutamic (GLU), 2-aminoadipic (2-AAA), threonine, aspartic, serine and asparagine were significantly higher in patients with T2DM compared with healthy controls. The concentration of 11 plasma acids, tyrosine, arginine (ARG), glycine (GLY), methionine, sarcosine, ornithine, lysine (LYS), glutamine (GLN) and three BCAAs, valine, leucine (LEU) and isoleucine, was significantly lower in patients with T2DM compared with healthy controls. By contrast, the levels of the other three amino acids, phenylalanine, proline (PRO) and hydroxyproline, were similar in both groups.

### Multiple regression analysis

Multiple logistic regression models, adjusted for age, gender and BMI, were applied to evaluate potential differences between patients with T2DM and healthy controls (p*-*values of age, gender and BMI were >0.05). Only plasma GLU and 2-AAA concentrations were significantly different between the groups, resulting in an OR of 1.113 (95% CI 1.006 to 1.231) for GLU and an OR of 9.027 (95% CI 1.059 to 76.926) for 2-AAA (Table 2). Plasma GLU and 2-AAA concentrations were not significantly correlated (r=−0.075, p=0.284).

**Table 2.** Multiple logistic regression between patients with T2DM (n=103) and healthy controls (n=104; reference).

Plasma acid	OR (95% CI)	p-value
Valine	0.983 (0.937 to 1.031)	0.474
Leucine	0.950 (0.888 to 1.016)	0.131
Isoleucine	0.960 (0.898 to 1.230)	0.253
Phenylalanine	0.982 (0.923 to 1.046)	0.575
Tyrosine	0.957 (0.837 to 1.093)	0.513
2-Aminoadipic	9.027 (1.059 to 76.926)	0.044
Arginine	0.872 (0.759 to 1.003)	0.055
Glycine	0.970 (0.940 to 1.001)	0.062
Threonine	1.055 (0.985 to 1.129)	0.127
Methionine	0.903 (0.609 to 1.340)	0.613
Aspatic	3.408 (0.354 to 32.792)	0.289
Sacosine	1.012 (0.997 to 1.027)	0.129
Ornithine	1.063 (0.885 to 1.276)	0.513
Proline	1.028 (0.993 to 1.064)	0.112
Lysine	0.893 (0.781 to 1.020)	0.096
Glutamic	1.113 (1.006 to 1.231)	0.037
Glutamine	0.960 (0.914 to 1.008)	0.101
Serine	1.072 (0.971 to 1.184)	0.169
Asparagine	1.017 (0.863 to 1.198)	0.844
Hydroxyproline	0.706 (0.456 to 1.093)	0.119

Abbreviation: T2DM, type 2 diabetes mellitus.

All values are presented as ORs (95% CI), adjusted for age, gender and body mass index.

### Subgroup analysis of patients with T2DM

**Figure 1.**
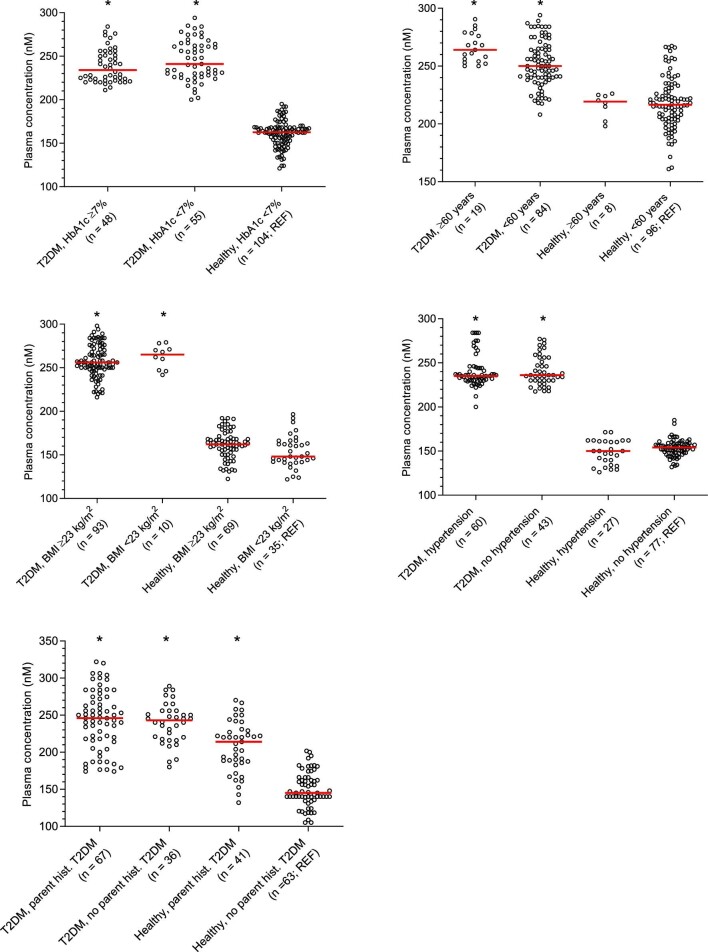
Scatter plots of plasma concentration of glutamic acids in patients with T2DM classified by HbA1c, age, BMI, hypertension and parental history of T2DM. *p<0.05 compared with the reference group.

To assess the association between amino acid profiles and important clinical characteristics in patients with T2DM, we performed subgroup analyses of patients with T2DM classified by five factors (HbA1c, age, BMI, hypertension and parental history of T2DM) using multinomial logistic regression. Plasma GLU concentrations were significantly higher in patients with T2DM compared with healthy subjects in all subgroups (Figure 1).

In the first subgroup, classified by plasma HbA1c (HbA1c ≥ 0.154 g/dl; uncontrolled T2DM), plasma GLU concentrations were significantly higher in patients with T2DM with both uncontrolled T2DM (OR=1.054, 95% CI 1.011 to 1.099) and controlled T2DM (OR=1.054, 95% CI 1.011 to 1.098) compared with healthy subjects as the reference group. Plasma ARG, LEU and GLN concentrations were significantly lower in patients with uncontrolled T2DM (OR=0.906, 95% CI 0.841 to 0.976; OR=0.931, 95% CI 0.876 to 0.989; and OR=0.976, 95% CI 0.958 to 0.995, respectively), but not in patients with controlled T2DM compared with healthy controls (Table S2).

In the second subgroup, classified by age ≥60 y, plasma GLU concentrations were significantly higher in patients with T2DM aged ≥60 y (OR=1.061, 95% CI 1.017 to 1.107) and <60 y (OR=1.056, 95% CI 1.012 to 1.102) compared with the reference group of healthy controls aged <60 y. Plasma LEU concentrations were significantly lower in all patients with T2DM (OR=0.931, 95% CI 0.870 to 0.995 in patients with T2DM aged ≥60 y and OR=0.939, 95% CI 0.885 to 0.996 in patients with T2DM aged <60 y) compared with the healthy controls group. Furthermore, plasma LYS concentrations were significantly lower in patients with T2DM aged ≥60 y (OR=0.907, 95% CI 0.841 to 0.978) but not in patients with T2DM aged <60 y compared with the healthy controls group (Table S3).

In the third subgroup, classified by overweight (BMI ≥23 kg/m^2^), plasma GLU concentrations were significantly higher in both overweight patients with T2DM (OR=1.063, 95% CI 1.019 to 1.110) and patients with T2DM at normal weight (OR=1.063, 95% CI 1.018 to 1.109) compared with the reference group of healthy subjects at normal weight. By contrast, plasma LEU concentrations were significantly lower in both patients with T2DM with overweight (OR=0.924, 95% CI 0.866 to 0.987) and normal weight (OR=0.888, 95% CI 0.813 to 0.970) compared with the healthy controls group. Furthermore, plasma concentrations of ARG (OR=0.907, 95% CI 0.837 to 0.984), GLY (OR=0.976, 95% CI 0.957 to 0.996) and GLN (OR=0.973, 95% CI 0.955 to 0.992) were significantly lower only in overweight patients with T2DM compared with the healthy controls group (Table S4).

In the fourth subgroup, classified by hypertension, plasma GLU concentrations were significantly higher in both patients with T2DM with hypertension (OR=1.053, 95% CI 1.011 to 1.096) and without hypertension (OR=1.056, 95% CI 1.014 to 1.099) compared with the reference group of healthy controls without hypertension. The plasma 2-AAA concentrations were significantly higher in subjects with hypertension for both patients with T2DM (OR=4.174, 95% CI 1.281 to 13.596) and healthy controls (OR=1.916, 95% CI 1.051 to 3.495) compared with the reference group. Also, plasma PRO concentrations were significantly higher in subjects with hypertension for both patients with T2DM (OR=1.043, 95% CI 1.008 to 1.079) and healthy controls (OR=1.031, 95% CI 1.009 to 1.054) compared with the reference group (Table S5). Furthermore, plasma concentrations of ARG (OR=0.898, 95% CI 0.832 to 0.970) and GLY (OR=0.975, 95% CI 0.953 to 0.998) were significantly lower in patients with T2DM with hypertension only compared with the reference group.

In the fifth subgroup, classified by parental history of T2DM, plasma GLU concentrations were significantly higher in patients with T2DM with a parental history of T2DM (OR=1.060, 95% CI 1.017 to 1.104), patients with T2DM without a parental history of T2DM (OR=1.060, 95% CI 1.018 to 1.104) and healthy controls with a parental history of T2DM (OR=1.012, 95% CI 1.002 to 1.023) compared with the reference group of healthy subjects without a parental history of T2DM.

### Correlation analysis

Plasma GLU concentrations in patients with T2DM were significantly correlated with plasma HbA1c (r=−0.23, p=0.019). However, plasma GLU concentration was not correlated significantly with age (r=0.037, p=0.711) or BMI (r=0.037, p=0.709).

Furthermore, plasma LEU concentrations were correlated significantly with plasma concentrations of ARG (r=0.350, p=0.001) and GLN (r=0.228, p=0.021). Plasma ARG was also correlated significantly with GLN (r=0.213, p=0.031). However, plasma LEU concentrations were not significantly correlated with HbA1c (r=−0.05, p=0.721) or BMI (r=0.054, p=0.601). Furthermore, neither plasma ARG nor GLN concentrations were correlated with HbA1c or BMI (r=0.050, p=0.738 for ARG and HbA1c; r=0.019, p=0.860 for ARG and BMI; r=−0.014, p=0.927 for GLN and HbA1c; and r=0.109, p=0.298 for GLN and BMI). There was no correlation between HbA1c and BMI (r=0.019, p=0.847).

## Discussion

Plasma GLU concentrations were significantly higher in patients with T2DM compared with healthy subjects (Tables 1 and 2). Plasma GLU was also significantly higher in all subgroups of patients with T2DM (Tables S2-S5). These results suggest that GLU was independently associated with T2DM and important clinical characteristics related to T2DM.

GLU is a non-essential amino acid (synthesized by most cells in the body) and a key intermediate of various metabolic pathways. It is derived by aminotransferase-mediated conversion of BCAAs. The metabolism of GLU and GLN is related to many cellular functions, including protein synthesis, insulin secretion in pancreatic β-cells and hepatic and renal gluconeogenesis for maintenance and promotion of cellular functions.^15^ GLN is a precursor for peptide and protein synthesis and also amino sugar synthesis. Furthermore, a common product of GLN metabolism is glutamate (anion of GLU), which is produced by glutaminase in most cells. However, GLN circulates in the blood and accumulates on the plasma membrane of β-cells, where it is converted to GLU.^16^

The metabolism of glutamate (anion of GLU) through glutamate dehydrogenase (GDH) in β-cells has an important function in the intracellular regulation of insulin. GDH is a mitochondrial matrix enzyme, which catalyzes the oxidative deamination of glutamate to α-ketoglutarate in a limited number of tissues in humans, including the pancreatic islets.^16^ Islet cells express functional glutamate receptors and vesicular glutamate transporters. α-ketoglutarate is a key intermediate in the Krebs cycle and glucose-mediated insulin secretion. Thus, the dysfunction of GLU metabolism has been related to insulin resistance and T2DM.

Previous studies have demonstrated that circulating levels of plasma GLU are associated with insulin resistance and an increased risk of T2DM,^17^ supporting our findings. A study in a Chinese population^18^ reported that increased GLU was related to the prevalence of T2DM. Higher glutamate levels were also associated with an increased risk of T2DM in a Spanish population (the PREDIMED trial).^19^ Furthermore, a Swedish study (the Malmö Preventive Project) showed that elevated plasma GLU was associated with future T2DM and coronary artery disease and that GLU was one of the most significant biomarkers of diabetic retinopathy (DR). Accumulation of glutamate in the retina caused neurotoxicity and DR development.^20^

We propose here that characterization of GLU profiles could be an effective biomarker for evaluating the risk of acquiring T2DM (a lifestyle-related disease) and that it could provide a better understanding of T2DM pathophysiology and enable early intervention to avoid disease progression and complication.

A limitation of this study is that a relatively small number of patients and controls were recruited at a single hospital in Thailand and therefore extrapolation to other populations should be undertaken with caution. Furthermore, diet, lifestyle and other clinical characteristics were not comparable between enrolled patients with T2DM and healthy controls, which might provide a bias in evaluation of the impact of amino acid profiles. The results presented here might not be generalizable to other Thai or Asian populations and these findings should be evaluated further and validated in large prospective cohort studies.

### Conclusions

The present analysis of amino acid profiles in patients with T2DM and healthy controls demonstrated that plasma GLU concentrations were independently associated with T2DM and correlated with important clinical characteristics related to T2DM. If validated in larger cohorts of patients, this could enhance our understanding of T2DM and its pathophysiology, as well as provide a biomarker for early intervention to avoid disease progression. This finding requires further evaluation.

## Supplementary Material

ihaa083_Supplemental_File

## Data Availability

The data underlying this article are available in the article and in its online supplementary material.
