# Supplementary material for: Assessment of the amino acid profile in Thai patients with type 2 diabetes mellitus using liquid chromatography-mass spectrometry
Source: Int Health. 2020 Oct 28;13(4):367–73. doi: 10.1093/inthealth/ihaa083 (PMC8253986; doi:10.1093/inthealth/ihaa083)
Supplement: ihaa083_Supplemental_File [file ihaa083_supplemental_file.docx]

# Supplementary Material

# Table S1. Ion transition, instrument settings and retention times for amino acids LC-MS detection.

|  | **Compound** | **Precursor (m/z)** | **Product (m/z)** | **DP (V)** | **CXP (V)** | **RT (min)** |
| --- | --- | --- | --- | --- | --- | --- |
| **Period 1** | Arginine | 303.4 | 69.90 | 41.00 | 14.00 | 2.70 |
|  | IS-Homoarginine | 317.3 | 84.10 | 41.00 | 4.000 | 2.90 |
|  | Glutamine | 275.3 | 172.1 | 26.00 | 10.00 | 3.30 |
|  | Serine | 234.3 | 146.0 | 101.0 | 8.000 | 3.70 |
|  | Asparagine | 243.3 | 157.2 | 31.00 | 10.00 | 3.80 |
|  | Hydroxyproline | 260.2 | 172.1 | 16.00 | 10.00 | 4.00 |
|  | Glycine | 204.1 | 144.0 | 21.00 | 8.000 | 4.30 |
|  | Threonine | 248.3 | 160.2 | 26.00 | 10.00 | 4.30 |
| **Period 2** | Sarcosine | 218.0 | 88.00 | 26.00 | 27.00 | 5.80 |
|  | Ornithine | 347.2 | 287.2 | 26.00 | 15.00 | 6.90 |
|  | IS-Methionine-d3 | 281.1 | 193.1 | 21.00 | 17.00 | 6.90 |
|  | Metionine | 278.3 | 190.1 | 21.00 | 17.00 | 7.00 |
|  | Proline | 244.3 | 156.2 | 26.00 | 19.00 | 7.10 |
|  | Lysine | 361.2 | 301.3 | 36.00 | 17.00 | 7.70 |
|  | Aspatic | 304.3 | 216.2 | 16.00 | 19.00 | 7.80 |
|  | Valine | 246.3 | 158.1 | 21.00 | 17.00 | 8.20 |
|  | Glutamic | 318.3 | 172.1 | 26.00 | 21.00 | 8.30 |
| **Period 3** | 2-Aminoadipic | 332.3 | 244.2 | 21.00 | 19.00 | 9.50 |
|  | Leucine | 260.3 | 172.1 | 26.00 | 17.00 | 10.00 |
|  | Phenylalanine | 294.3 | 206.1 | 26.00 | 17.00 | 10.10 |
|  | Isoleucine | 260.3 | 172.1 | 26.00 | 17.00 | 10.60 |
|  | IS-Homophenylalanine | 308.3 | 220.2 | 36.00 | 15.00 | 12.20 |
|  | Tyrosine | 396.2 | 136.2 | 46.00 | 43.00 | 13.30 |

† Abbreviations; DP = declustering potential, CXP = collision cell exit potential and RT = retention time.

‡ Period 1 at RT = 0.00-5.00 min, Period 2 at RT = 5.01-9.00 min and Period 3 at RT = 9.01-17.00 min.

# Table S2. Multinomial logistic regression between T2DM patients with controlled and uncontrolled HbA1c, compared with healthy subjects as a reference group.

|  | **T2DM with controlled**  **HbA1c (n = 55)** | | **T2DM with uncontrolled HbA1c (n = 48)** | |
| --- | --- | --- | --- | --- |
| **Plasma acid** | **Odds Ratio**  **(95% CI)** | ***P* value** | **Odds Ratio**  **(95% CI)** | ***P* value** |
| Isoleucine | 1.035 (0.991-1.082) | 0.123 | 1.047 (1.000-1.095) | 0.051 |
| Valine | 0.986 (0.961-1.010) | 0.249 | 0.988 (0.963-1.013) | 0.337 |
| Leucine | 0.942 (0.888-1.000) | 0.051 | 0.931 (0.876-0.989) | **0.021** |
| Phenylalanine | 0.988 (0.951-1.025) | 0.515 | 0.979 (0.943-1.017) | 0.272 |
| Tyrosine | 0.988 (0.914-1.069) | 0.770 | 1.018 (0.943-1.100) | 0.647 |
| 2-Aminoadipic | 2.052 (0.669-6.288) | 0.209 | 2.136 (0.696-6.558) | 0.185 |
| Arginine | 0.932 (0.867-1.002) | 0.058 | 0.906 (0.841-0.976) | **0.009** |
| Glycine | 0.984 (0.968-1.001) | 0.063 | 0.988 (0.971-1.005) | 0.151 |
| Threonine | 1.011 (0.982-1.041) | 0.475 | 1.009 (0.980-1.040) | 0.539 |
| Methionine | 1.123 (0.871-1.449) | 0.371 | 1.082 (0.838-1.397) | 0.547 |
| Aspatic | 1.797 (0.633-5.101) | 0.271 | 1.593 (0.546-4.644) | 0.394 |
| Sacosine | 1.001 (0.990-1.012) | 0.870 | 0.996 (0.985-1.008) | 0.537 |
| Ornithine | 1.025 (0.938-1.120) | 0.588 | 1.009 (0.921-1.105) | 0.853 |
| Proline | 1.026 (0.997-1.056) | 0.084 | 1.015 (0.985-1.047) | 0.318 |
| Lysine | 0.950 (0.890-1.014) | 0.123 | 0.970 (0.908-1.036) | 0.362 |
| Glutamic | 1.054 (1.011-1.098) | **0.013** | 1.054 (1.011-1.099) | **0.012** |
| Glutamine | 0.982 (0.965-1.000) | 0.050 | 0.976 (0.958-0.995) | **0.011** |
| Serine | 1.050 (0.985-1.120) | 0.134 | 1.059 (0.993-1.130) | 0.080 |
| Asparagine | 0.966 (0.881-1.059) | 0.463 | 0.947 (0.863-1.040) | 0.255 |
| Hydroxyproline | 0.901 (0.744-1.091) | 0.286 | 0.919 (0.758-1.114) | 0.391 |

All values are presented as odds ratios (95% confidence interval).

† Abbreviations; HbA1c = Hemoglobin A1c, T2DM = type 2 Diabetes Mellitus, 95% CI = confidential interval.

‡ T2DM with controlled HbA1c (n = 55) was defined as patients with plasma HbA1c < 7%, T2DM with uncontrolled HbA1c (n = 48) was defined as patients with HbA1c ≥ 7%, and the reference group was defined as healthy subjects with HbA1c < 7% (n = 104).

# Table S3. Multinomial logistic regression between T2DM patients above and below 60 years of age, compared with healthy subjects as a reference group.

|  | | | | | | |  |  |  |  |
| --- | --- | --- | --- | --- | --- | --- | --- | --- | --- | --- |
|  | **Healthy ≥ 60 years**  **(n = 8)** | **T2DM < 60 years**  **(n = 84)** | | | | **T2DM ≥ 60 years**  **(n = 19)** | | | |  |
| **Plasma acid** | **Odds Ratio**  **(95% CI)** | ***P* value** | **Odds Ratio**  **(95% CI)** | ***P* value** | **Odds Ratio**  **(95% CI)** | | | ***P value*** | |  |
|  |  |  |  |  |  | | |  | | |
| Isoleucine | 1.003 (0.961-1.047) | 0.891 | 1.039 (0.994-1.086) | 0.089 | 1.042 (0.993-1.094) | | | 0.096 |  |  |
| Valine | 1.007 (0.985-1.030) | 0.553 | 0.982 (0.958-1.007) | 0.162 | 0.995 (0.967-1.024) | | | 0.724 | |  |
| Leucine | 0.983 (0.936-1.032) | 0.491 | 0.939 (0.885-0.996) | **0.035** | 0.931 (0.870-0.995) | | | **0.035** | |  |
| Phenylalanine | 1.012 (0.973-1.054) | 0.545 | 0.985 (0.949-1.024) | 0.449 | 0.984 (0.946-1.024) | | | 0.432 | |  |
| Tyrosine | 1.007 (0.896-1.130) | 0.913 | 0.995 (0.923-1.073) | 0.896 | 0.976 (0.885-1.075) | | | 0.618 | |  |
| 2-Aminoadipic | 1.077 (0.474-2.447) | 0.860 | 2.324 (0.722-7.476) | 0.157 | 1.316 (0.307-5.633) | | | 0.712 | |  |
| Arginine | 1.020 (0.969-1.073) | 0.451 | 0.928 (0.861-1.000) | 0.051 | 0.928 (0.857-1.004) | | | 0.063 | |  |
| Glycine | 1.000 (0.993-1.007) | 0.990 | 0.983 (0.966-1.002) | 0.073 | 0.981 (0.960-1.002) | | | 0.072 | |  |
| Threonine | 0.977 (0.941-1.014) | 0.223 | 1.010 (0.980-1.041) | 0.518 | 1.020 (0.988-1.053) | | | 0.223 | |  |
| Methionine | 0.883 (0.659-1.184) | 0.405 | 1.080 (0.842-1.385) | 0.543 | 1.224 (0.924-1.620) | | | 0.159 | |  |
| Aspatic | 0.686 (0.267-1.763) | 0.434 | 1.612 (0.548-4.739) | 0.385 | 1.618 (0.524-4.997) | | | 0.403 | |  |
| Sacosine | 1.002 (0.989-1.014) | 0.809 | 1.002 (0.991-1.013) | 0.753 | 1.000 (0.987-1.012) | | | 0.957 | |  |
| Ornithine | 1.031 (0.965-1.101) | 0.371 | 1.027 (0.938-1.125) | 0.564 | 1.073 (0.973-1.185) | | | 0.159 | |  |
| Proline | 1.013 (0.986-1.041) | 0.335 | 1.028 (0.997-1.061) | 0.076 | 1.014 (0.979-1.050) | | | 0.437 | |  |
| Lysine | 0.991 (0.952-1.032) | 0.663 | 0.951 (0.891-1.016) | 0.135 | 0.907 **(**0.841-0.978) | | | **0.011** | |  |
| Glutamic | 1.010 (0.993-1.028) | 0.248 | 1.056 **(**1.012-1.102) | **0.011** | 1.061 (1.017-1.107) | | | **0.006** | |  |
| Glutamine | 0.997 (0.977-1.016) | 0.738 | 0.980 (0.961-0.998) | 0.301 | 0.986 (0.965-1.008) | | | 0.211 | |  |
| Serine | 1.011 (0.970-1.054) | 0.600 | 1.057 (0.987-1.133) | 0.113 | 1.051 (0.980-1.127) | | | 0.166 | |  |
| Asparagine | 1.012 (0.914-1.120) | 0.818 | 0.964 (0.874-1.063) | 0.460 | 0.927 (0.832-1.033) | | | 0.170 | |  |
| Hydroxyproline | 0.972 (0.872-1.083) | 0.603 | 0.879 (0.720-1.074) | 0.207 | 0.896 (0.732-1.097) | | | 0.288 | |  |

All values are presented as odds ratios (95% confidence interval).

† Abbreviations; T2DM = type 2 Diabetes Mellitus, 95% CI = confidence interval.

‡ Healthy ≥ 60 years was defined as healthy subjects ≥ 60 years of age (n = 8), T2DM < 60 years was defined as T2DM patients < 60 years of age (n = 84), T2DM ≥ 60 years was defined as T2DM patients ≥ 60 years of age (n = 19), and the reference group was defined as healthy subjects < 60 years of age (n = 96).

# Table S4. Multinomial logistic regression between T2DM patients at overweight and normal weight with overweight, compared with healthy subjects as a reference group.

|  | | | | | | |  |  |
| --- | --- | --- | --- | --- | --- | --- | --- | --- |
|  | **Healthy overweight**  **(n = 69)** | | **T2DM normal weight**  **(n = 10)** | | **T2DM overweight**  **(n = 93)** | | | |
| **Plasma acid** | **Odds Ratio (95% CI)** | **p value** | **Odds Ratio (95% CI)** | **p value** | **Odds Ratio (95% CI)** | ***p value*** | |  |
| Isoleucine | 1.004 (0.983-1.024) | 0.728 | 1.070 (1.003-1.143) | 0.401 | 1.045 (0.996-1.097) | 0.073 | |  |
| Valine | 1.010 (0.996-1.024) | 0.179 | 0.982 (0.939-1.026) | 0.414 | 0.988 (0.959-1.018) | 0.428 | |  |
| Leucine | 0.982 (0.958-1.006) | 0.146 | 0.888 **(**0.813-0.970) | **0.008** | 0.924 (0.866-0.987) | **0.018** | |  |
| Phenylalanine | 1.014 (0.994-1.034) | 0.177 | 1.016 (0.967-1.067) | 0.527 | 0.994 (0.953-1.037) | 0.793 | |  |
| Tyrosine | 1.043 (0.984-1.105) | 0.153 | 1.008 (0.904-1.124) | 0.885 | 1.033 (0.946-1.129) | 0.469 | |  |
| 2-Aminoadipic | 0.997 (0.623-1.594) | 0.990 | 0.595 (0.057-6.201) | 0.664 | 2.444 (0.699-8.548) | 0.162 | |  |
| Arginine | 1.001 (0.979-1.023) | 0.933 | 0.935 (0.860-1.016) | 0.113 | 0.907 (0.837-0.984) | **0.019** | |  |
| Glycine | 0.996 (0.992-1.000) | 0.060 | 0.989 (0.971-1.007) | 0.227 | 0.976 (0.957-0.996) | **0.016** | |  |
| Threonine | 1.007 (0.994-1.021) | 0.288 | 1.017 (0.977-1.058) | 0.411 | 1.016 (0.981-1.052) | 0.376 | |  |
| Methionine | 1.025 (0.890-1.181) | 0.732 | 1.084 (0.767-1.532) | 0.648 | 1.175 (0.880-1.569) | 0.274 | |  |
| Aspatic | 1.206 (0.746-1.950) | 0.445 | 0.661 (0.160-2.738) | 0.568 | 1.885 (0.646-5.501) | 0.246 | |  |
| Sacosine | 1.001 (0.993-1.008) | 0.878 | 0.998 (0.981-1.015) | 0.835 | 1.002 (0.989-1.016) | 0.76 | |  |
| Ornithine | 1.000 (0.970-1.032) | 0.994 | 1.000 (0.890-1.123) | 1.000 | 1.026 (0.922-1.143) | 0.635 | |  |
| Proline | 0.996 (0.984-1.009) | 0.540 | 1.024 (0.983-1.065) | 0.253 | 1.028 (0.992-1.066) | 0.129 | |  |
| Lysine | 0.988 (0.968-1.008) | 0.235 | 0.974 (0.907-1.046) | 0.473 | 0.940 (0.879-1.004) | 0.066 | |  |
| Glutamic | 1.005 (0.995-1.015) | 0.351 | 1.063 (1.018-1.109) | **0.006** | 1.063 (1.019-1.110) | **0.005** | |  |
| Glutamine | 0.997 (0.989-1.006) | 0.494 | 0.987 (0.966-1.009) | 0.237 | 0.973 (0.955-0.992) | **0.005** | |  |
| Serine | 1.008 (0.989-1.028) | 0.397 | 1.065 (0.994-1.142) | 0.074 | 1.065 (0.996-1.140) | 0.067 | |  |
| Asparagine | 0.974 (0.932-1.018) | 0.244 | 0.915 (0.822-1.018) | 0.103 | 0.955 (0.865-1.055) | 0.365 | |  |
| Hydroxyproline | 0.995 (0.947-1.045) | 0.833 | 0.825 (0.649-1.049) | 0.116 | 0.888 (0.725-1.088) | 0.253 | |  |

All values are presented as odds ratios (95% confidence interval).

† Abbreviations; BMI = body mass index, T2DM = type 2 Diabetes Mellitus, 95% CI = confidence interval.

‡ Healthy overweight was defined as healthy subjects with BMI ≥ 23 kg/m^2^ (n = 69), T2DM normal weight was defined as T2DM patients with BMI < 23 kg/m^2^ (n = 10), T2DM overweight was defined as T2DM patients ≥ 23 kg/m^2^ (n = 93), and the reference group was defined as healthy subjects at normal weight (BMI < 23 kg/m^2^; n = 35).

# Table S5. Multinomial logistic regression between T2DM patients with and without hypertension, compared with healthy subjects as a reference group.

|  | **Healthy hypertension**  **(n = 27)** | | **T2DM no hypertension**  **(n = 43)** | | **T2DM hypertension**  **(n = 60)** | |
| --- | --- | --- | --- | --- | --- | --- |
| **Plasma acid** | **Odds Ratio**  **(95% CI)** | ***p* value** | **Odds Ratio**  **(95% CI)** | ***p* value** | **Odds Ratio**  **(95% CI)** | ***p* value** |
| Isoleucine | 0.997 (0.973-1.022) | 0.807 | 1.030 (0.977-1.085) | 0.281 | 1.031 (0.980-1.084) | 0.236 |
| Valine | 1.003 (0.982-1.025) | 0.768 | 0.985 (0.955-1.015) | 0.328 | 0.993 (0.963-1.023) | 0.632 |
| Leucine | 0.996 (0.966-1.028) | 0.823 | 0.942 (0.878-1.010) | 0.094 | 0.939 (0.875-1.007) | 0.078 |
| Phenylalanine | 1.035 (1.003-1.067) | 0.030 | 0.990 (0.952-1.030) | 0.623 | 0.992 (0.955-1.031) | 0.683 |
| Tyrosine | 1.086 (0.996-1.185) | 0.062 | 0.994 (0.907-1.088) | 0.890 | 1.018 (0.931-1.112) | 0.699 |
| 2-Aminoadipic | 1.916 (1.051-3.495) | **0.034** | 2.049 (0.590-7.114) | 0.259 | 4.174 (1.281-13.596) | **0.018** |
| Arginine | 0.988 (0.958-1.019) | 0.445 | 0.927 (0.859-1.000) | 0.051 | 0.898 (0.832-0.970) | **0.006** |
| Glycine | 0.996 (0.990-1.001) | 0.108 | 0.987 (0.967-1.007) | 0.211 | 0.975 (0.953-0.998) | **0.031** |
| Threonine | 0.999 (0.981-1.016) | 0.891 | 1.013 (0.979-1.048) | 0.455 | 1.018 (0.984-1.053) | 0.303 |
| Methionine | 0.766 (0.610-0.962) | 0.022 | 1.083 (0.815-1.439) | 0.582 | 1.084 (0.811-1.450) | 0.586 |
| Aspatic | 0.376 (0.172-0.822) | 0.014 | 1.049 (0.333-3.303) | 0.935 | 1.263 (0.395-4.044) | 0.694 |
| Sacosine | 1.001 (0.991-1.011) | 0.881 | 0.999 (0.986-1.012) | 0.836 | 1.009 (0.996-1.023) | 0.177 |
| Ornithine | 0.970 (0.927-1.015) | 0.194 | 1.017 (0.920-1.124) | 0.744 | 1.000 (0.906-1.104) | 0.993 |
| Proline | 1.031 (1.009-1.054) | **0.006** | 1.026 (0.989-1.064) | 0.169 | 1.043 (1.008-1.079) | **0.016** |
| Lysine | 1.018 (0.991-1.045) | 0.189 | 0.965 (0.900-1.034) | 0.314 | 0.972 (0.906-1.042) | 0.420 |
| Glutamic | 1.014 (0.999-1.029) | 0.065 | 1.056 (1.014-1.099) | **0.009** | 1.053 (1.011-1.096) | **0.012** |
| Glutamine | 1.008 (0.995-1.022) | 0.221 | 0.980 (0.953-1.008) | 0.157 | 0.979 (0.951-1.007) | 0.139 |
| Serine | 1.006 (0.983-1.028) | 0.630 | 1.050 (0.978-1.127) | 0.181 | 1.053 (0.981-1.131) | 0.154 |
| Asparagine | 0.975 (0.919-1.035) | 0.406 | 0.977 (0.829-1.151) | 0.782 | 0.941 (0.799-1.108) | 0.467 |
| Hydroxyproline | 1.029 (0.970-1.092) | 0.345 | 0.884 (0.722-1.082) | 0.231 | 0.918 (0.753-1.119) | 0.398 |

All values are reported as odds ratios (95% confidence interval).

† Abbreviations; T2DM = type 2 Diabetes Mellitus, 95% CI = confidence interval.

‡ Healthy hypertension was defined as healthy subjects with hypertension (n = 69), T2DM no hypertension was defined as T2DM patients with normal blood pressure (n = 43), T2DM hypertension was defined as T2DM patients with hypertension (n = 60), and the reference group was defined as healthy subjects at with normal blood pressure (n = 77). Hypertension was defined as systolic blood pressure ≥ 140 mmHg and/or diastolic blood pressure ≥ 90 mmHg.
